# SnS_2_ Nanosheets as a Template for 2D SnO_2_ Sensitive Material: Nanostructure and Surface Composition Effects

**DOI:** 10.3390/ma15228213

**Published:** 2022-11-18

**Authors:** Roman Vasiliev, Darya Kurtina, Nataliya Udalova, Vadim Platonov, Abulkosim Nasriddinov, Tatyana Shatalova, Roman Novotortsev, Xiaogan Li, Marina Rumyantseva

**Affiliations:** 1 Chemistry Department, Moscow State University, 119991 Moscow, Russia; 2 Faculty of Materials Science, Moscow State University, 119991 Moscow, Russia; 3 School of Microelectronics, Key Lab of Liaoning for Integrated Circuits Technology, Dalian University of Technology, Dalian 116024, China

**Keywords:** 2D nanomaterials, template synthesis, SnS_2_ nanosheets, SnO_2_ nanosheets, gas sensor, carbon monoxide, ammonia

## Abstract

Two-dimensional nanosheets of semiconductor metal oxides are considered as promising for use in gas sensors, because of the combination of a large surface-area, high thermal stability and high sensitivity, due to the chemisorption mechanism of gas detection. In this work, 2D SnO_2_ nanosheets were synthesized via the oxidation of template SnS_2_ nanosheets obtained by surfactant-assisted one-pot solution synthesis. The 2D SnO_2_ was characterized using transmission and scanning electron microscopy (TEM, SEM), X-ray diffraction (XRD), low-temperature nitrogen adsorption, X-ray photoelectron spectroscopy (XPS) and IR spectroscopy. The sensor characteristics were studied when detecting model gases CO and NH_3_ in dry (RH_25_ = 0%) and humid (RH_25_ = 30%) air. The combination of high specific-surface-area and increased surface acidity caused by the presence of residual sulfate anions provides a high 2D SnO_2_ sensor’s signal towards NH_3_ at a low temperature of 200 °C in dry air, but at the same time causes an inversion of the sensor response when detecting NH_3_ in a humid atmosphere. To reveal the processes responsible for sensor-response inversion, the interaction of 2D SnO_2_ with ammonia was investigated using diffuse reflectance infrared Fourier transform spectroscopy (DRIFTS) in dry and humid air at temperatures corresponding to the maximum “positive” and maximum “negative” sensor response.

## 1. Introduction

Two-dimensional (2D) semiconductor nanomaterials have attracted a great deal of research interest due to their unique dimension-dependent electronic properties. Two-dimensional semiconductors may find a variety of applications, such as high-mobility transistors [1] and sensitive photodetectors [2], as well as gas sensors [3,4]. The family of 2D materials significantly increased in 2010–2020, and now includes materials that are diverse in nature: phosphorene, an analog of graphene consisting of atomically thin layers of phosphorus [5,6]; a group of materials with the common name MXenes, including 2D carbides, nitrides and carbonitrides [7,8]; boron nitride [9]; molybdenum, tungsten and rhenium dichalcogenides [10,11]; layered semiconductor chalcogenides GaS, GaSe, SnS_2_ [12,13,14] and layered oxides (MoO_3_) [15]. It should be noted that for practical application in the field of gas sensors, 2D materials such as graphene and its derivatives or MoS_2_, MoSe_2_, WS_2_, WSe_2_ have a number of significant limitations [16,17,18]. The main disadvantage of 2D materials is their low stability and fully saturated surface. Temperature rise and thermal cycling in air, to clean the surface of layered chalcogenides, phosphorene, and carbides (MXenes) leads to surface oxidation, degradation of adsorption properties and increased resistance. A common disadvantage of 2D materials is their reasonably low sensitivity due to the primary contribution of physical adsorption instead of chemisorption into the sensor signal.

The 2D nanosheets of semiconductor metal oxides may be considered as promising for use in gas sensors, since the combination of a large surface-area, high thermal stability and high sensitivity, due to the chemisorption mechanism of gas detection, is expected. However, the semiconductor oxides ZnO, SnO_2_, In_2_O_3_, WO_3_, most commonly used as gas-sensitive materials, do not have a layered structure, which makes it difficult to synthesize them in the form of 2D nanosheets by traditional methods such as the exfoliation route. For example, various methods of hydrothermal synthesis have been proposed to obtain SnO_2_ [19,20,21,22,23,24,25,26], but they allow the acquirement only of hierarchical 3D structures (of the nanoflowers type) formed by 2D nanosheets. An interesting example is the synthesis of 2D indium oxide, InO, in the spatial confinement between SiC and graphene using the MOCVD method [27]. It was found that the predicted atomic arrangement of intercalated In atoms is consistent with the In–In distances predicted for the stable 2D InO structure [28]. The authors of [28] also reported on the structural and electronic properties of In_2_O_3_ 2D structures with planar hexagonal geometry.

An alternative approach may be template synthesis, in which 2D nanosheets of layered sulfide are oxidized to form an oxide while preserving the morphology of the particles. The recent works in this direction are the articles by V. Paolucci et al. [29,30], presenting SnSe_2_ 2D nanosheets as a template for obtaining amorphous SnO_2_ layers. 

Tin disulfide SnS_2_, a widely known layered material, is an indirect semiconductor with an energy gap of approximately 2.07 eV [31]. It has attracted interest in photovoltaic [32], photocatalytic [33,34] and sensor [35] applications and also as a material for Li-ion batteries [36,37]. Recently, SnS_2_ nanoparticles with a morphology of nanoflowers [33,34] or nanosheets [36,37] were synthesized using surfactant-assisted hydrothermal routes. Growth of SnS_2_ nanoplatelets in nonpolar solutions using the solvotermal method starting from SnCl_4_·5H_2_O [32] or by the thermal decomposition of Sn(S_2_CNEt_2_)_4_ single-molecular precursor [38] with the use of oleylamine as the surfactant, was reported.

Here, we report a simple method of producing 2D SnO_2_ nanosheets via the oxidation of template SnS_2_ nanosheets obtained by surfactant-assisted one-pot solution synthesis. The 2D SnO_2_ was characterized in detail, in terms of microstructure parameters and surface composition, and also tested as a gas-sensitive material when detecting CO and NH_3_.

## 2. Materials and Methods

All chemicals were purchased from Sigma Aldrich in the purest form available and used for the syntheses without further purification. 

Colloidal SnS_2_ nanosheets were synthesized under argon atmosphere, following the protocol outlined here. Tin (IV) acetate Sn(CH_3_COO)_4_ (0.023 mmol) and oleic acid (0.09 mmol) were added to 1-octadecene (2 mL) followed by heating at 200 °C for approximately 1 h under argon flow to remove the acetic acid and form the tin oleate complex. The solution was then cooled to room temperature, and dodecylamine (0.09 mmol) and elemental sulfur (0.045 mmol) were added. The mixture was heated again to 220 °C under vigorous stirring and held at this temperature for approximately 5 min. As the desired temperature was reached, the initial colorless solution changed to yellow and then to turbid orange. The resultant solution was cooled, mixed with an equal volume of acetone, and centrifuged at 6000 rpm for 10 min. The supernatant was discarded, and the sediment nanosheets were redispersed in toluene. The SnS_2_ nanosheets were additionally precipitated using an equal volume of acetone, separated by centrifugation, and redispersed in the toluene. The resultant orange dispersion was slightly turbid and was stable to aggregation for several days.

**Figure 1:**
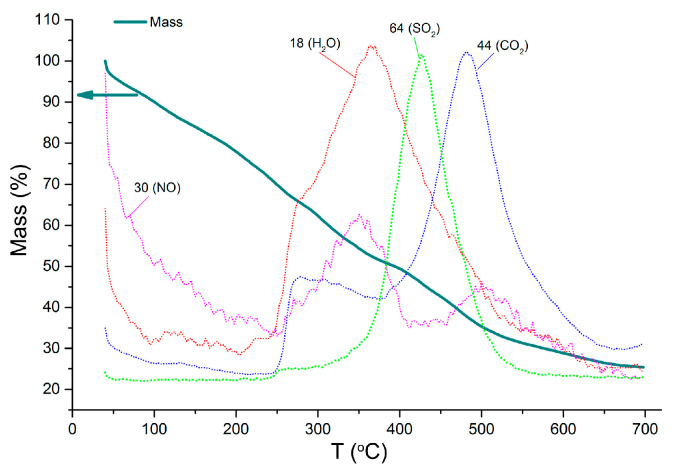
TG curve and temperature dependencies of ionic current for *m*/*z* = 18 (H_2_O), 30 (NO), 44 (CO_2_), 64 (SO_2_) for SnS_2_ oxidation in air.

The conditions of complete SnS_2_ to SnO_2_ oxidative transformation were determined by thermogravimetric analysis with mass spectral analysis of gaseous products (TG-MS), using a NETZSCH STA 409 PC/PG instrument (heating in air, 5K/min) (Figure 1). The oxidation of organic stabilizers (oleic acid and dodecylamine) occurs in the temperature range of 250–600 °C, as evidenced by a symbiotic increase in ionic currents, corresponding to the mass numbers *m*/*z* = 18 (H_2_O), 44 (CO_2_) and 30 (NO). The oxidation of sulfide anions, which is accompanied by the release of SO_2_ (*m*/*z* = 64) occurs in a narrower temperature range of 250–550 °C. To obtain 2D SnO_2_, the SnS_2_ sol was dried in air at room temperature until the solvent was removed. The SnS_2_ powder was annealed at 500 °C for 6 h. The annealing temperature of 500 °C was chosen to obtain 2D SnO_2_ with high specific-surface- area.

The size and morphology of the SnS_2_ nanoparticles were determined by transmission electron microscopy (TEM), with the LEO19 AB OMEGA microscope operated at 100 kV and the JEOL JEM2100 microscope operated at 200 kV. The morphology of the SnS_2_ and 2D SnO_2_ powders was studied with scanning electron microscopy (SEM), using a Carl Zeiss SUPRA 40 FE-SEM instrument with Inlens SE detector (accelerating voltage 5 kV, aperture 30 μm). The specific surface area of 2D SnO_2_ was measured by the low-temperature nitrogen adsorption, using a Chemisorb 2750 instrument (Micromeritics).

The phase composition was analyzed using X-ray diffraction (XRD) and Raman spectroscopy. A Panalytical Aeris Research diffractometer (CuKα radiation, Bragg–Brentano geometry, PiXCel detector, with a total angular range of 3.000–60.000° 2θ, a step size of ca. 0.005° and variable exposure time) was used for X-ray-powder-diffraction measurements. For this investigation, concentrated solutions of the purified nanocrystals were spread on top of a silicon wafer. The SnO_2_ crystallite size was estimated using the Scherrer formula. Raman spectra of SnS_2_ were acquired on a Renishaw InVia Raman microscope equipped with a 514 nm argon laser. Raman spectra of 2D SnO_2_ were collected on the i-Raman Plus spectrometer (BW Tek) equipped with a BAC 151C microscope and a 532 nm laser. 

The surface composition was analyzed using X-ray photoelectron spectroscopy (XPS) and Fourier transform infrared (FTIR) spectroscopy. XP spectra were obtained on Omicron ESCA+ (monochromatic AlKα anode, E = 1486.6 eV) using a neutralizer (scanning step 0.1 eV/s, transmission energy 20 eV). The spectra were processed using the UNIFIT software. The peaks were approximated by convolution of the Gauss and Lorentz functions, with the simultaneous optimization of the background parameters. The FTIR spectra were registered in transmission mode using a Frontier (Perkin Elmer) spectrometer in the 4000–400 cm^−1^ region, with a step of 1 cm^−1^. For these experiments, 0.3–0.5 mg of the powder was ground with 40 mg KBr (FT-IR grade, Sigma-Aldrich, St. Louis, MO, USA) and pressed into tablets (~0.5 mm thick, 12 mm in diameter). The baseline was preliminarily taken from pure KBr. 

Diffuse reflectance infrared fourier-transformed (DRIFT) spectra were recorded on a Frontier (Perkin Elmer) spectrometer using a DiffusIR annex and heated-flow chamber HC900 (Pike Technologies, Fitchburg, WI, USA) sealed by a ZnSe window. DRIFT spectra were registered in the 4000–1000 cm^−1^ region with resolution 4 cm^−1^ and with accumulation of 30 scans with automatic H_2_O/CO_2_ compensation. The 2D SnO_2_ powder (30 mg) was placed in alumina crucibles (5 mm diameter). The measurements were performed under 100 mL/min flow of dry (relative humidity at 25 °C RH_25_ = 0%) or humid (RH_25_ = 30%) air, containing 100 ppm NH_3_ at 200 °C and at 350 °C. 

**Figure 2:**
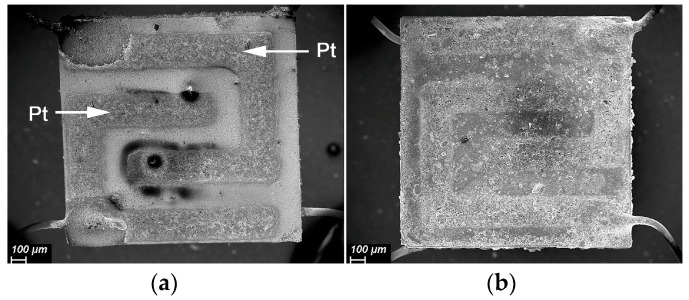
SEM image of alumina substrate with Pt contacts: (**a**) bare substrate, (**b**) covered with 2D SnO_2_.

To manufacture the 2D SnO_2_ sensors, concentrated SnS_2_ sol in toluene was deposited dropwise on the alumina substrates with Pt contacts and Pt heaters (Figure 2). To form a sensitive film, the deposition of SnS_2_ was repeated three times. After each deposition, the layer was dried in air at room temperature until the solvent evaporated, and then slowly heated to 500 °C, using a substrate heater. To oxidize SnS_2_ into 2D SnO_2_, the formed films were additionally annealed in air at 500 °C for 6 h. The selected annealing temperature of 500 °C allows for complete oxidation of the SnS_2_, and provides the necessary thermal stability of 2D SnO_2_ during sensor measurements in the temperature range of 50–500 °C. Certified gas mixtures containing 2530 ppm CO in N_2_ and 240 ppm NH_3_ in N_2_ were used as gas sources. The concentration of the target gas in the air was set and controlled using EL-FLOW mass-flow controllers (Bronkhorst). The flow rate through the measuring cell in all measurements was constant 100 ± 0.5 mL/min. The humidity of the gas mixture (relative humidity at 25 °C RH_25_ = 0% for dry conditions and RH_25_ = 30% for humid conditions) was set and controlled by a P-2 Humidifier (Cellkraft). The sensor resistance was measured at 1.3 V DC-voltage at a temperature fixed in the range of 50–500 °C, with the step of 50 °C. For each temperature, three cycles of measurements (15 min in pure air, 15 min in the presence of air containing the target gas), were performed. The sensor response was calculated as S = (G_gas_ − G_air_)/G_air_, where G_air_ is the sensor conductance in air, and G_gas_ is the sensor conductance in the CO- or NH_3_-containing gas mixture.

## 3. Results

### 3.1. Microstructure, Phase Composition and Surface Composition

**Figure 3:**
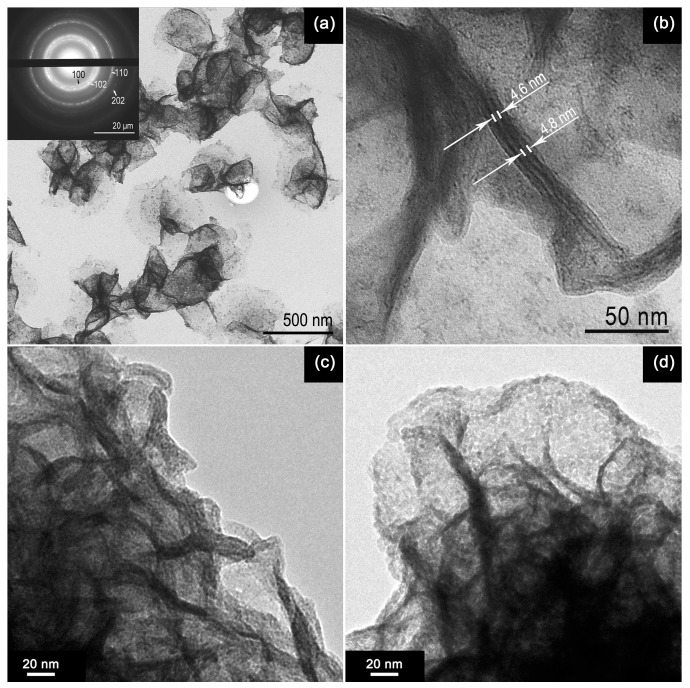
TEM images of SnS_2_ nanosheets before (**a**–**c**) and after (**d**) oxidation at 500 °C. Inset shows the electron-diffraction pattern.

**Figure 4:**
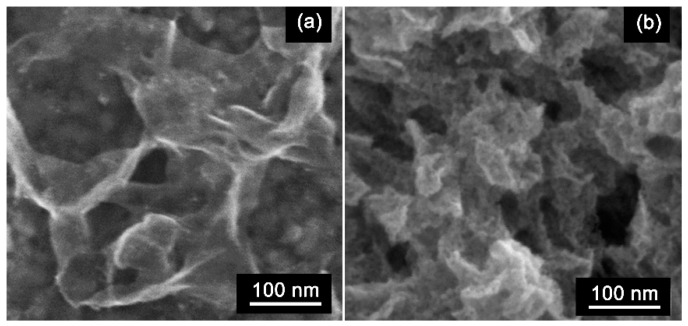
SEM images of SnS_2_ nanosheets before (**a**) and after (**b**) oxidation at 500 °C.

The nanosheet morphology was studied using the transmission and scanning electron microscopy (TEM and SEM) methods. Figure 3 shows a series of representative TEM images of the SnS_2_ sample. The SnS_2_ nanosheets have 2D morphology. The sample is formed by~ 5 nm thick nanosheets packed in agglomerates of 4–5 pieces (Figure 3b). The electron-diffraction pattern (inset in Figure 3a) taken from an ensemble of nanosheets shows that particles have the crystal structure of SnS_2_ (berndtite). When oxidized at 500 °C, the structure of the 2D nanosheets is mostly preserved. (Figure 3c,d). SEM images (Figure 4) reveal the formation of nanosheets with lateral lengths > 500 nm. 

**Figure 5:**
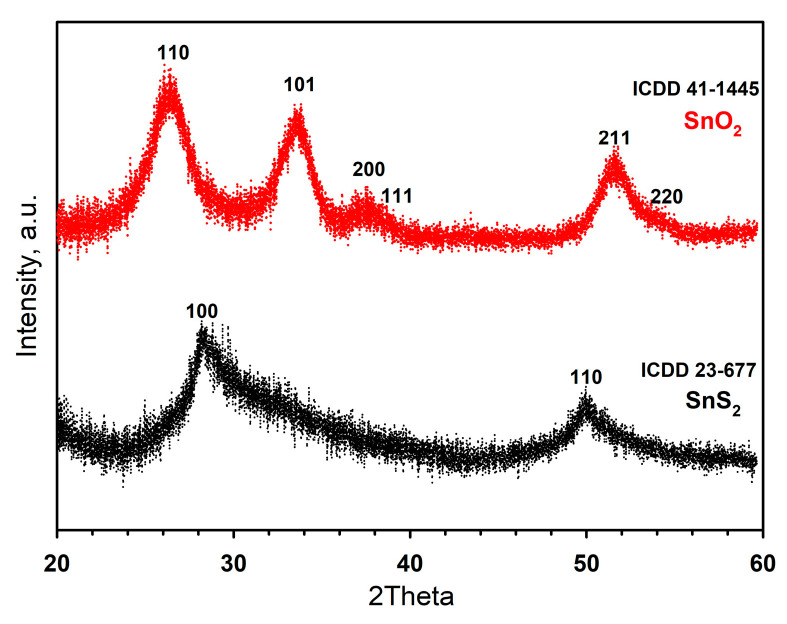
X-ray diffraction patterns of SnS_2_ nanosheets and 2D SnO_2_ powder.

The X-ray diffraction pattern of the SnS_2_ nanosheets (Figure 5) is close to the diffractogram of the SnS_2_ Berndtite-2T standard (ICDD No. 23-677). The diffraction pattern contains broadened reflections (100) and (110), which refer to the directions lying in the plane of the sheet. At the same time, the reflection (001) corresponding to the normal to the plane of the sheet does not appear. This may be due to the extremely small thickness of the sheet that indicates the implementation of a 2D structure. The oxidation of the SnS_2_ nanosheets leads to the formation of SnO_2_ with a cassiterite structure (ICDD No. 41-1445). The diffraction maxima of 2D SnO_2_ are greatly broadened, which indicates the small size of the crystal grains. The crystallite size of the 2D SnO_2_ calculated using Sherrer’s formula with (110) and (101) reflections is 4.0 ± 0.5 nm, which correlates with the high specific area of 44 ± 2 m^2^/g. 

**Figure 6:**
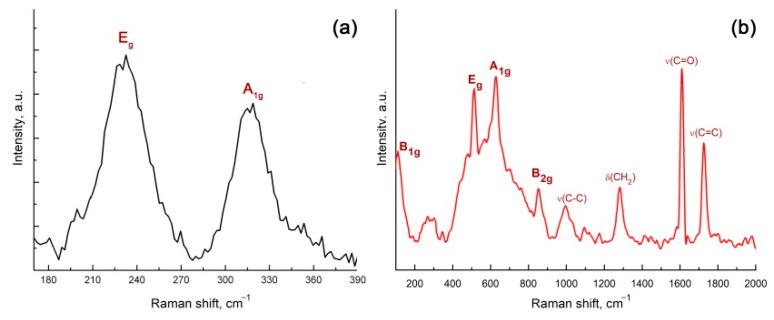
Raman spectra of SnS_2_ (**a**) and 2D SnO_2_ (**b**) samples.

Figure 6 compares the Raman spectra of the SnS_2_ and 2D SnO_2_ nanosheets. Assignments of Raman vibrational modes are presented in Table 1. Two peaks with maxima at 230 cm^−1^ and 317 cm^−1^ are observed in the Raman spectrum of the SnS_2_ nanosheets (Figure 6a). The latter corresponds to the A_1g_ mode of SnS_2_ [39]. The line at 230 cm^−1^ is shifted towards large wavenumbers compared with E_g_ SnS_2_ (205–210° cm^−1^ for a single crystal [39,40]) and is very intense, compared with A_1g_. Such modification of the spectrum may be due to the dimensional effect and the formation of a 2D structure [41]. In the Raman spectrum of the 2D SnO_2_ sample (Figure 6b), there are A_1g_, E_g_, B_2g_, B_1g_ tin dioxide modes as well as a wide band in the range of 400–700 cm^−1^, corresponding to the superposition of surface modes [42]. The assignment of Raman bands corresponding to volume modes is made on the basis of the literature data [43]. The appearance of surface modes is associated with the small size of the SnO_2_ particles, and may be due to the manifestation of symmetry-forbidden oscillations, due to a disturbance of the long-range order in systems of reduced dimension [42]. An alternative explanation is the formation of a highly defective near-surface layer, the contribution of which is maximal for materials with the smallest particle size [44]. In the range of 900–2000 cm^−1^ in the spectrum of 2D SnO_2_, there are lines corresponding to the residues of the oleic acid stabilizer used in the synthesis of the SnS_2_ nanosheets (Table 1).

**Figure 7:**
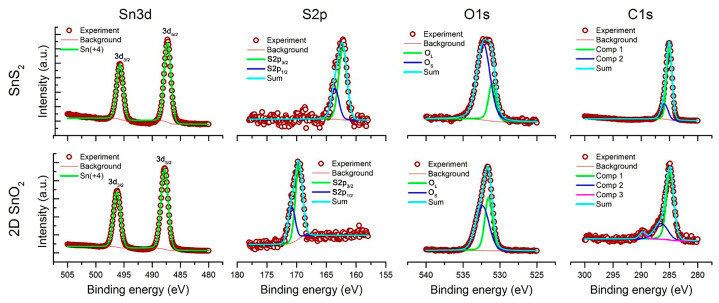
XP-spectra of SnS_2_ and 2D SnO_2_.

XP-spectra SnS_2_ and 2D SnO_2_ are shown in Figure 7. In both cases, tin is present in the oxidation state +4. The positions of the S2p lines correspond to the sulfide anion (162.3 eV, 163.5 eV) in the case of SnS_2_, and the sulfate anion (169.6 eV, 170.8 eV) in the case of 2D SnO_2_. The ratio of the integral intensities of the sulfur and tin signals is [S]/[Sn] = 0.24 and 0.04 for SnS_2_ and 2D SnO_2_, respectively. This indicates that in 2D SnO_2_, sulfates are surface residues. The O1s spectra contain two components corresponding to oxygen of the oxide (O_L_, 530.7–531.2 eV) and oxygen from the surface oxygen-containing species (O_S_, 532.0–532.4 eV). The ratio of the integral intensities of oxygen and tin signals [O]/[Sn] = 0.37 and 0.24 for SnS_2_ and 2D SnO_2_, respectively. Such a large amount of oxygen from oxygen-containing particles on the SnS_2_ surface is due to the presence of an organic stabilizer, oleic acid. The C1s spectrum is also complex, and contains two or three components corresponding to the presence of C-C bonds (C1, 284.8–285.0 eV), C-OH and C-O-C bonds (C2, 285.9–286.5 eV) and carboxyl groups COO^−^ (C3, 289.1–289.8 eV). The C3 component appears only in the 2D SnO_2_ spectrum. The ratio of the carbon and tin signal-intensities is [C]/[Sn] = 2.22 and 0.04 for SnS_2_ and 2D SnO_2_, respectively. The main part of the carbon on the surface of the SnS_2_ is obviously represented by a long-chain hydrocarbon radical of oleic acid.

**Figure 8:**
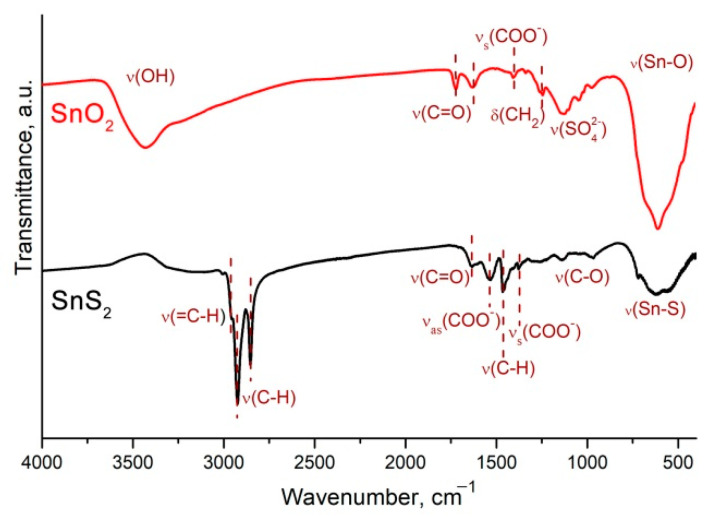
FTIR spectra of SnS_2_ and 2D SnO_2_ samples.

The FTIR spectra shown in Figure 8 are consistent with the results of the Raman spectroscopy and XPS. The assignment of absorption bands is presented in Table 1 [45]. There are many hydrocarbon fragments and carboxylic groups which may be attributed to oleic acid ligands covering the SnS_2_ surface. On the surface of the 2D SnO_2_, only residues of the organic-stabilizer oxidation products and sulfate anions were found. 

### 3.2. Gas Sensor Properties

**Figure 9:**
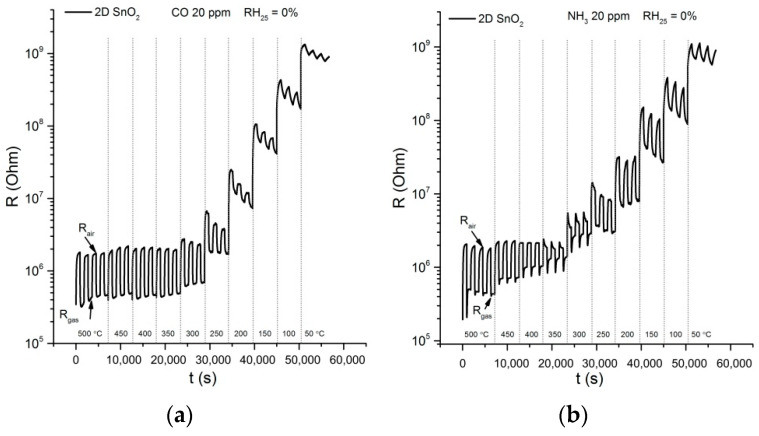
Temperature dependencies of the dynamic-sensor response of 2D SnO_2_ when detecting 20 ppm CO (**a**) and 20 ppm NH_3_ (**b**) in dry air (RH_25_ = 0%).

To evaluate the sensor properties of the 2D SnO_2_ model, reducing gases CO (which does not have specific acid-base properties) and NH_3_ (which exhibits basic properties) were selected. The measurements were carried out when detecting 20 ppm CO and 20 ppm NH_3_ in dry (RH_25_ = 0%) and humid (RH_25_ = 30%) air. Examples of changes in the resistance of the 2D SnO_2_ when detecting 20 ppm CO and 20 ppm NH_3_ in dry air (RH_25_ = 0%) are shown in Figure 9. The sensor’s resistance decreases in the presence of reducing, due to their oxidation by oxygen chemisorbed on the SnO_2_ surface:

(1) $${\mathrm{CO}}_{\left(\mathrm{gas}\right)}+O_{\beta \left(\mathrm{ads}\right)}^{\alpha -}\to {\beta \mathrm{CO}}_{2\left(\mathrm{gas}\right)}+{\alpha e}^{-}$$

(2) $$2{\mathrm{NH}}_{3\left(\mathrm{gas}\right)}+\frac{3}{\beta }O_{\beta \left(\mathrm{ads}\right)}^{\alpha -}\to N_{2\left(\mathrm{gas}\right)}+3H_{2}O_{\left(\mathrm{gas}\right)}+\frac{3\alpha }{\beta }e^{-}$$

where ${\mathrm{CO}}_{\left(\mathrm{gas}\right),\;}{\mathrm{NH}}_{3\left(\mathrm{gas}\right)}$ are CO and NH_3_ molecules in the gas phase, $O_{\beta \left(\mathrm{ads}\right)}^{-\alpha }$ is the chemisorbed oxygen species (α = 1 and 2 for once- and twice-charged particles, respectively; β = 1 and 2 for atomic and molecular forms, respectively), e^−^ is an electron injected into the conduction band, and ${\mathrm{CO}}_{2\left(\mathrm{gas}\right)},N_{2\left(\mathrm{gas}\right)},{\;H}_{2}O_{\left(\mathrm{gas}\right)}$ are products of CO and NH_3_ oxidation desorbed into the gas phase. 

In the temperature range 350–500 °C, the independence of the 2D SnO_2_ resistance in air on the measurement temperature is observed. Such an unusual type of temperature dependence of the semiconductor resistance may be due to the small thickness of the sensitive layer formed from the sol during the oxidation of the SnS_2_ nanosheets. Therefore, the surface of all 2D SnO_2_ particles is accessible for oxygen chemisorption, which occurs with electron localization. As a result, the 2D SnO_2_ particles with a thickness of several nanometers turn out to be completely depleted of electrons. This corresponds to the situation of the “flat zones”, with the same concentration of electrons in the bulk and near the surface of the crystallite [46], in which the barriers at the grain boundaries that determine the value of the conductivity activation-energy are small. With a decrease in the operating temperature, the thickness of the depleted layer near the 2D SnO_2_ surface decreases. This leads to a difference in the electron concentration in the bulk and near the crystallite surface, which leads to the formation of significant surface barriers and a transition to the activation character of conductivity.

At temperatures below 200 °C, baseline drift (the change in resistance in air at the same operating temperature) is observed. This effect may be due to incomplete desorption and accumulation of the products of CO and NH_3_ oxidation on the surface of sensitive material.

**Figure 10:**
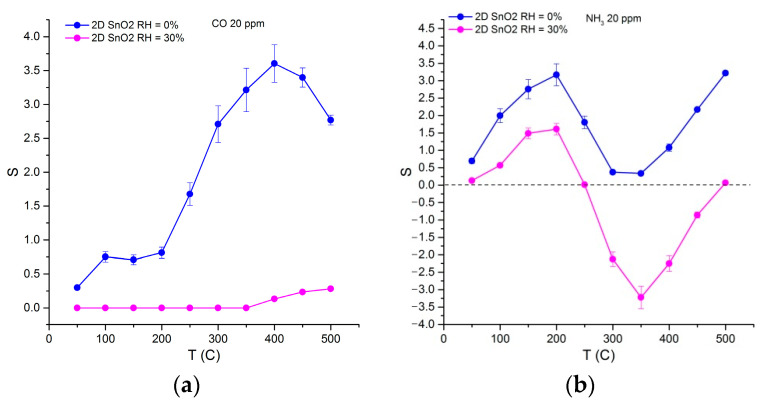
Temperature dependencies of 2D SnO_2_ sensor response when detecting 20 ppm CO (**a**) and 20 ppm NH_3_ (**b**) in dry (RH_25_ = 0%) and humid (RH_25_ = 30%) air.

The temperature dependencies of the sensor’s response are shown in Figure 10. When detecting CO (Figure 10a), there is a significant decrease in the response value. Such a change in signal is apparently because of a decrease in the number of oxidative active-centers on the SnO_2_ surface, namely, chemisorbed oxygen anions, which are responsible for the formation of a sensor response when detecting CO [47]. In humid air, dissociative adsorption of water vapor leads to the substitution of both lattice and chemisorbed oxygen by hydroxyl groups [48]. It can be expected that for thin 2D SnO_2_ sensitive layers formed from the sol during the oxidation of the SnS_2_ nanosheets, the process of surface hydroxylation in humid air occurs to a high extent. 

The temperature dependencies of the sensor’s response towards NH_3_ have a more complex form (Figure 10b). In dry air, a maximum sensor signal is observed at T = 200 °C. The surface sulfate anions act as additional acid centers, favored for NH_3_ adsorption [49]. The temperature range of 250–350 °C corresponds to the minimum sensor response, and a further temperature increase leads to an increase in the sensor signal. When NH_3_ is detected in moist air, the sensor response of 2D SnO_2_ acquires “negative” values in this temperature range, due to the fact that the resistance of this material in the presence of ammonia becomes greater than in pure air. 

Such a change in the response type was reported for various materials: from *n*- to *p*-type for MoO_3_ [50], In_2_O_3_ [51], SnO_2_ [52,53,54], SnO_2_(Fe) [55], SnO_2_(Pd,Pt) [56], ZnO [57], WO_3_ [58,59], TiO_2_ [60], and from *p*- to *n*-type conductivity for α-Fe_2_O_3_ [61], Co_3_O_4_ [62], graphene [63,64], and SnO_2_- and WO_3_-decorated graphene [65]. The inversion of the sensor response was explained by different reasons: (i) a change in the type of main charge-carriers in the semiconductor oxide, due to either the surface reactions under certain conditions, or because of the effect of impurities; (ii) kinetic reasons related to the adsorption barrier of the detected gas; (iii) the formation of new donor or acceptor species, which contributes to the change in the sensor conductivity.

The results obtained in this work and in our previous article [54] allow us to conclude that when detecting ammonia, the most likely cause of signal inversion is precisely the appearance of new acceptor species. The reason for the decrease in the SnO_2_ response in the temperature range of 250–350 °C is the possible NH_3_ to NO oxidation by chemisorbed oxygen. Further interaction of NO molecules with ambient oxygen molecules leads to the formation of surface-bound nitrite and nitrate groups [66]. This process occurs with the localization of charge carriers from the conduction band of the semiconductor, which leads to a decrease in the electrical conductivity of the *n*-type semiconductor material and a formal decrease in the sensor response. The adsorption of water vapor on the SnO_2_ surface occurs with an increase in the electron concentration in the conduction band of the semiconductor [48]. This should stimulate the formation of surface nitrite and nitrate groups which occurs with the localization of electrons, and cause a greater increase in the resistance of the sensitive layer. 

### 3.3. In Situ DRIFTS Analysis of 2D SnO_2_ Interaction with NH_3_

**Figure 11:**
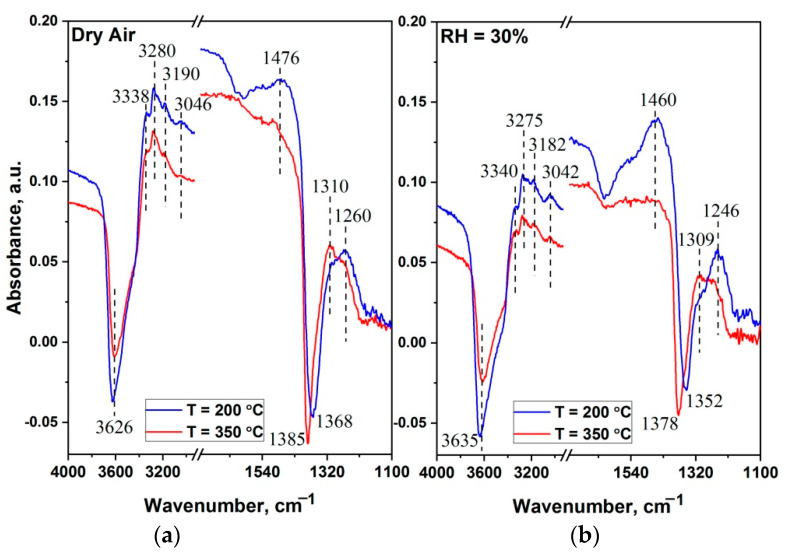
In situ DRIFT spectra of 2D SnO_2_ (**a**) in dry (RH_25_ = 0%) and (**b**) in humid (RH_25_ = 30%) air containing 100 ppm NH_3_ at temperatures of 200 °C (blue line) and 350 °C (red line).

To confirm the above reasoning, the interaction of the 2D SnO_2_ with ammonia was investigated using DRIFTS in dry (RH_25_ = 0%) and humid (RH_25_ = 30%) air at temperatures of 200 °C and 350 °C, corresponding to the maximum "positive" and maximum "negative" sensor response, respectively. The DRIFT spectra recorded after 100 min exposure in dry or humid air containing 100 ppm NH_3_ are shown in Figure 11. Spectra in dry and humid air at 200 °C and 350 °C were used as the baselines.

In general, the spectra obtained at 200 °C and 350 °C in dry and humid air are similar (Table 2), but there are also differences corresponding to the type of change in conductivity. It can be assumed that the adsorption of ammonia molecules proceeds to a greater extent on the Brønsted acid active-sites, which results in a decrease in the intensity of the υ(OH) and δ(H_2_O) groups, the protonation of NH_3_ and the appearance of bands corresponding to the bending vibrations of NH_4_^+^ at 1460 and 1476 cm^−1^. The bands in the range of 1246–1260 cm^−1^ are associated with vibrations of the NH_3_^+^ coordinated at the Lewis acid sites (tin ions), while the N-H stretching-vibration region of coordinated NH_3_ is revealed in the form of many narrow peaks in the range of 3042–3340 cm^−1^ [67,68].

At 200 °C, the presence of intense peaks related to NH_3_^+^ and NH_4_^+^ assumes that ammonia is the main reagent interacting with the surface of the sensitive layer of the sensor. Comparing the intensities of these peaks, one can see that a humid atmosphere is a more favorable condition for the adsorption of NH_3_ species, at both the Brønsted and Lewis acid sites. At the same time, at 350 °C, a decrease in the intensity of peaks related to the NH_3_ species and the appearance of an additional peak at 1310 cm^−1^ can be noticed. The latter corresponds either to chelating bidentate nitrate or chelating bidentate nitrite groups [43,44,69]. The presence of nitrate groups on the surface at high temperature conditions may indicate the oxidation of ammonia molecules to NO (process (3)), and then, with the participation of ambient oxygen, further conversion to NO_2_ (processes (4) and (5)). As was shown earlier by Wang et al. [70], the maximum conversion of NO to NO_2_ in NH_3_-pre-adsorbed samples is achieved in the temperature range of 350–400 °C.

(3) $$2{\mathrm{NH}}_{3\left(\mathrm{gas}\right)}+\frac{5}{\beta }O_{\beta \left(\mathrm{ads}\right)}^{\alpha -}\to 2{\mathrm{NO}}_{\left(\mathrm{ads}\right)}+3H_{2}O_{\left(\mathrm{gas}\right)}+\frac{5\alpha }{\beta }e^{-}$$

(4) $$2{\mathrm{NO}}_{\left(\mathrm{ads}\right)}+O_{2\left(\mathrm{gas}\right)}+2e^{-}\to 2{\mathrm{NO}}_{2\left(\mathrm{ads}\right)}^{-}$$

(5) $$2{\mathrm{NO}}_{2\left(\mathrm{ads}\right)}^{-}+O_{2\left(\mathrm{gas}\right)}\to 2{\mathrm{NO}}_{3\left(\mathrm{ads}\right)}^{-}$$

Processes (4) and (5) can lead to a decrease in the conductivity of the sample, and might be the main reason for the reversing of the sensor signal (Figure 10b). Our results are in agreement with the observations of Zhou et al. [68] and Ramis et al. [71], who observed NH_3_ over-oxidation to NO or N_2_ at high temperatures above 300 °C.

The appearance of negative IR-adsorption-bands after NH_3_ adsorption at the range of 1352–1385 cm^−1^, depending on the sample, is directly related to the presence of sulfate groups on the surface [43,44]. Hadjiivanov et al. have reported the formation of a negative band in this region, and explained it by the adsorption of species in the immediate vicinity of sulfate anions [72]. Ramis et al. have attributed the negative band at 1362 cm^−1^ to the perturbation of the S=O band of traces of sulphate impurities in γ-Fe_2_O_3_ [71].

However, it should be noted that this negative absorption-peak also appears during the interaction of ammonia with the sample with pre-adsorbed SO_2_. Thus, according to Zhang et al., the formation of the negative band at 1368 cm^−1^ is associated with the replacement of the sulfate species by the ammonia ones, while the interaction of SO_2_ with the NH_3_ pre-treated sample leads to the production of (NH_4_)_2_SO_4_ or NH_4_HSO_4_ [67].

## 4. Conclusions

In summary, we have developed a new route to obtain two-dimensional SnO_2_ for sensor applications. The 2D SnO_2_ nanosheets were successfully synthesized via the oxidation (at 500 °C) of SnS_2_ nanosheets obtained in solution during the simple chemical reaction. TEM and SEM data confirmed that the as-grown SnS_2_ nanosheets and oxidized SnO_2_ nanosheets have 2D morphology. The 2D SnO_2_ is characterized by a high specific-surface area and increased surface-acidity, caused by the presence of residual sulfate anions. The combination of these characteristics provides a high 2D SnO_2_ sensor signal towards NH_3_ at a low temperature of 200 °C in dry air, but at the same time causes an inversion of the sensor response when detecting NH_3_ in a humid atmosphere. We have to conclude that SnS_2_ nanosheets are a good template for synthesizing 2D SnO_2_. Moreover, the features of the active surface-centers formed may be useful when creating sensors for a particular gas with pronounced basic properties. We believe that synthesized 2D SnO_2_ will be interesting for the design of new sensitive materials with high sensor performances.

## Figures and Tables

**Table 1:** Assignments of IR bands and Raman vibrational modes (cm^−1^).

Sample	IR Band/Region, cm^−1^	Assignment	Raman Shift, cm^−1^	Assignment
SnS_2_	400–800	ν (Sn-S) + ν (Sn-O)	230	E_g_
	970, 1130	ν (C-O)	317	A_1g_
	1372	ν_s_ (COO^−^)		
	1462, 2852, 2924	ν (C-H)		
	1534	ν_as_ (COO^−^)		
	1633	ν (C=O)		
	2955	ν (=C-H)		
2D SnO_2_	400–800	ν (Sn-O)	110.5	B_1g_
	970, 1048, 1122	ν (SO_4_^2−^)	270	IR E_u_
	1255	δ (CH_2_)	513.5	E_g_
	1400	νs (COO^−^)	564.8	surface mode
	1626	δ (H_2_O) + ν (C=O)	627	A_1g_
	1728	ν (C=O)	851.7	B_2g_
	2850–3700	“free” OH groups	997.5	ν (C-C)
			1282.6	δ (CH_2_)
			1609.6	ν (C=O)
			1727.7	ν (C=C)

**Table 2:** Assignments of IR absorption bands (cm^−1^) appeared in DRFIT spectra on the surface of 2D SnO_2_ in dry (RH_25_ = 0%) and in humid (RH_25_ = 30%) air containing 100 ppm NH_3_ at 200 °C and 350 °C.

Functional Groups	200 °C		350 °C	
	RH_25_ = 0%	RH_25_ = 30%	RH_25_ = 0%	RH_25_ = 30%
NH_3_^+^ on Lewis acid site	1258	1246	1268	1260
chelating bidentate nitrate (NO_3_^−^) or nitrite (NO_2_^−^)	-	-	1310	1309
SO_2_ in (-SO_2_∙NH-) or SO_4_^2−^	1368	1352	1385	1378
NH_4_^+^ on Brønsted acid site	1476	1460	-	-
δ(H_2_O)	1620	1625	-	1625
ν(N-H) in NH_4_^+^	3046	3042	3046	3042
ν(N-H) in NH_3_	3185, 3280	3184, 3270	3190, 3280	3189, 3278
ν(OH)	3330–3770	3330–3780	3330–3730	3330–3780

## Data Availability

The data that support the findings of this study are available from the corresponding author upon reasonable request.
